# Engineered Production of Short Chain Fatty Acid in *Escherichia coli* Using Fatty Acid Synthesis Pathway

**DOI:** 10.1371/journal.pone.0160035

**Published:** 2016-07-28

**Authors:** Kamran Jawed, Anu Jose Mattam, Zia Fatma, Saima Wajid, Malik Z. Abdin, Syed Shams Yazdani

**Affiliations:** 1 Microbial Engineering Group, International Centre for Genetic Engineering and Biotechnology, Aruna Asaf Ali Marg, New Delhi, India; 2 Department of Biotechnology, Jamia Hamdard, Hamdard Nagar, New Delhi, India; 3 DBT-ICGEB Centre for Advanced Bioenergy Research, International Centre for Genetic Engineering and Biotechnology, Aruna Asaf Ali Marg, New Delhi, India; University of Houston, UNITED STATES

## Abstract

Short-chain fatty acids (SCFAs), such as butyric acid, have a broad range of applications in chemical and fuel industries. Worldwide demand of sustainable fuels and chemicals has encouraged researchers for microbial synthesis of SCFAs. In this study we compared three thioesterases, i.e., TesAT from *Anaerococcus tetradius*, TesBF from *Bryantella formatexigens* and TesBT from *Bacteroides thetaiotaomicron*, for production of SCFAs in *Escherichia coli* utilizing native fatty acid synthesis (FASII) pathway and modulated the genetic and bioprocess parameters to improve its yield and productivity. *E*. *coli* strain expressing *tesBT* gene yielded maximum butyric acid titer at 1.46 g L^-1^, followed by *tesBF* at 0.85 g L^-1^ and *tesAT* at 0.12 g L^-1^. The titer of butyric acid varied significantly depending upon the plasmid copy number and strain genotype. The modulation of genetic factors that are known to influence long chain fatty acid production, such as deletion of the *fadD* and *fadE* that initiates the fatty acid degradation cycle and overexpression of *fadR* that is a global transcriptional activator of fatty acid biosynthesis and repressor of degradation cycle, did not improve the butyric acid titer significantly. Use of chemical inhibitor cerulenin, which restricts the fatty acid elongation cycle, increased the butyric acid titer by 1.7-fold in case of TesBF, while it had adverse impact in case of TesBT. *In vitro* enzyme assay indicated that cerulenin also inhibited short chain specific thioesterase, though inhibitory concentration varied according to the type of thioesterase used. Further process optimization followed by fed-batch cultivation under phosphorous limited condition led to production of 14.3 g L^-1^ butyric acid and 17.5 g L^-1^ total free fatty acid at 28% of theoretical yield. This study expands our understanding of SCFAs production in *E*. *coli* through FASII pathway and highlights role of genetic and process optimization to enhance the desired product.

## Introduction

Short chain fatty acids (SCFAs), such as butyric acid (C4), are promising intermediates of many chemical and biofuel molecules. For example, butyric acid is a precursor for short chain fatty alcohols like butanol, which is superior to ethanol in terms of energy density, vapor pressure and hygroscopicity, and is a direct replacement of gasoline [1, 2]. Short chain alkanes like propane, with highly favorable physicochemical properties as a fuel and a major component of liquefied petroleum gas has been produced in *E*. *coli* via butyric acid as an intermediate [3, 4]. Short chain fatty alkyl esters (SCFAEs), also known as volatile esters, produced by esterification of short chain acid and alcohol, occur naturally in many ripening fruits and flowers and have broad application in the flavor, fragrance, cosmetic, solvent, paint and coating industries [5–7]. In addition to these, butyric acid also has therapeutic applications as its derivatives are used for the treatment of diseases like cancer, sickle cell anemia and alopecia [5, 8, 9].

Currently butyric acid is produced on industrial scale by chemical synthesis from crude oils that involves the oxidation of butyraldehyde. It can also be extracted from butter as its concentration in butter ranges from 2% to 4%. However, these chemical means to produce butyric acid is costly and non-ecofriendly [5, 9, 10]. Therefore, there is a need to produce butyric acid from renewable carbon sources using a microbial platform to replace its chemical synthesis.

**Fig 1:**
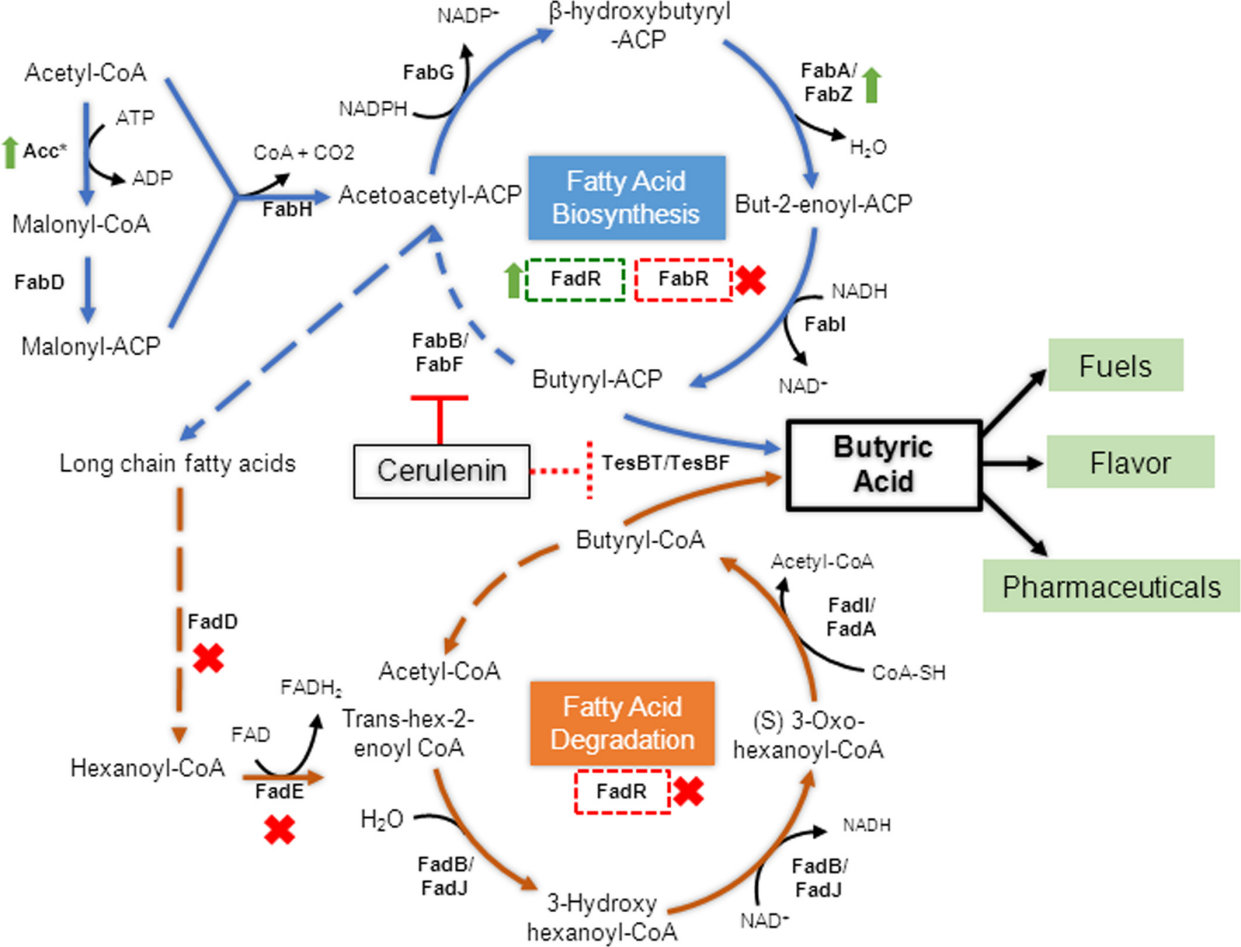
Schematic representation of various strategies adopted to engineer *E*. *coli* for production of butyric acid. Fatty acid synthesis and degradation cycles have been depicted with blue and orange arrows, respectively. Regulatory proteins have been shown in dotted box in green and red color denoting activation and repression, respectively. Genes that have been overexpressed in this study have been denoted with green upward arrow, while genes that have been deleted are shown along with red cross. Abbreviations: TesAT—thioesterase of *Anaerococcus tetradius*; TesBT–thioesterase of *Bacteroides thetaiotaomicron*; TesBF—thioesterase of *Bryantella formatexigens*; Acc*—acetyl-CoA carboxylase of *Corynebacterium glutamicum*; FabD–malonyl CoA-ACP transacylase; FabH - β- ketoacyl-ACP synthase III; FabG - β-ketoacyl-ACP reductase; FabA and FabZ - β-hydroxyacyl-ACP dehydratase; FabI—enoyl-ACP reductase; FabB - β-ketoacyl-ACP synthase I; FadD—fatty acyl-CoA synthetase; FadE—acyl CoA dehydrogenase; FabR–transcriptional repressor of FASII; FadR–transcriptional enhancer of FASII and repressor of β-oxidation.

Butyric acid production by microbial fermentation has been investigated with natively producing microbes such as *Clostridium tyrobutyricum*, *C*. *beijerinckii*, and *C*. *thermobutyricum* [11]. However, these microbes are strictly anaerobic and spore forming in nature, and their ability to produce butyric acid in specific conditions, such as narrow pH window, high ATP concentration, low NADH/NAD+ ratio, etc., demanded development of another versatile platform [8]. *E*. *coli* as an industrial workhorse for production of many bio-based chemicals becomes the natural choice. Since *E*. *coli* does not natively produce butyric acid, one could either transfer complete fermentative pathway from anaerobes into *E*. *coli* and thus restricting its growth to anaerobic condition, or exploit aerobically active fatty acid synthesis cycle (Fig 1). Engineering *E*. *coli* fatty acid biosynthesis (FASII) pathway is a promising avenue to produce fatty acid-derived fuels and chemicals [12]. Significant progress has been made in engineering this pathway for production of medium and long-chain FFAs. For example, medium-chain FFAs C12 and C14 were produced endogenously by heterologously expressed thioesterase BTE from *Umbellularia californica* in *E*. *coli* [13] and their subsequent conversion to the corresponding alcohols have also been reported [14]. *E*. *coli* strains carrying the thioesterase genes from *Ricinus communis* and *Jatropha curcas* accumulated more than 2.0 g L^-1^ FFAs of chain length C14, C16:1 and C16 during 48 h fermentation [11]. To date, much less attention has been paid to produce SCFAs in *E*. *coli* through FASII pathway [15, 16]. A maximum titer of ~ 0.44 g L^-1^ for butyric acid was achieved so far though this pathway [3]. There is a report of production of unsaturated SCFA, i.e., butenoic acid, using FASII pathway with titer of 4 g L^-1^ after 48 hr of fed-batch fermentation [16] and through reversal of β-oxidation pathway 500 mg L^-1^ 3-ketobutyric acid, 150 mg L^-1^ 3-hydroxybutyric and 200 mg L^-1^ of trans-2-butenoic acid has been reported [17]. There has also been effort to import fermentative pathway from *Clostridium* sp into *E*. *coli* in the form of synthetic gene scaffolds, which resulted in production of 7.2 g L^-1^ of butyric acid under fed-batch fermentation [2]. In second case, instead of thioesterase, the authors expressed *atoDA* to convert acetate and butyryl-CoA to butyrate and acetyl-CoA and achieved 10 g L^-1^ of butyric acid; however 8 g L^-1^ acetic acid had been supplemented in the glucose feed to reach to this titer [10].

Recently, Jing et al. [18] analyzed several thioesterases from different organisms and shown their ability to produce different chain length fatty acids. We selected three thioesterases, i.e., TesAT from *Anaerococcus tetradius*, TesBT from *Bacteroides thetaiotaomicron* and TesBF from *Bryantella formatexigens*, for their ability to produce short chain fatty acids, overexpressed them in *E*. *coli* and analyzed role of host fatty acid synthesis pathway and its regulatory network in production of butyric acid. We further performed process optimization and shown highest level of butyric acid titer of 14.3 g L^-1^ reported by engineered *E*. *coli* so far by fed-batch cultivation.

## Materials and Methods

### Microbial strains, reagents and media

*E*. *coli* strains used in this study have been listed in Table 1. *E*. *coli* DH5α was used as host for molecular cloning. Gene deletions in the production host were achieved through P1 phage transduction method [19, 20] using the single gene knockout strains from Coli Genetic Stock Centre (CGSC), Yale University, USA. The kanamycin resistant marker gene was removed by a helper plasmid pCP20 expressing the flippase (FLP) and the resultant strain was used for sequential rounds of gene knockout. Deletion was confirmed via PCR using primers towards flanking regions of the gene, as mentioned in Table 1.

**Table 1:** Strains, plasmids and primers used in this study.

Name	Description	Reference
***E*. *coli* Strains**		
MG1655	F-, λ-, rph-1	CGSC #6300
JM109	endA1 glnV44 thi-1 relA1 gyrA96 recA1 mcrB+ Δ(lac-proAB) e14- [F' traD36 proAB+ lacIq lacZΔM15] hsdR17(rK-mK+)	Promega
*E*. *coli* B	F-	CGSC #2507
DH5α	F- Φ80lacZΔM15 Δ(lacZYA-argF) U169 recA1 endA1 hsdR17	Invitrogen
M15	F- thi lac mtl, pREP4 plasmid	Qiagen
BW25113	rrnB DElacZ4787 HsdR514 DE(araBAD)567DE(rhaBAD)568 rph-1	CGSC#7636
BL21 (DE3)	F- ompT gal dcm lon hsdSB(rB−mB−) λ(DE3)	Invitrogen
MG1655 ∆*fadD*	MG1655, Δ*fadD*:: FRT-kan-FRT	This Study
MG1655 ∆*fadE*	MG1655, Δ*fadE*:: FRT-kan-FRT	This Study
MG1655 ∆*fadD*∆*fadE*	MG1655, Δ*fadD* Δ*fadE*:: FRT-kan-FRT	This Study
MG1655 ∆*fabR*	MG1655, Δ*fabR*:: FRT-kan-FRT	This Study
**Plasmids**		
pQE30	ColE1 ori T5 Lac O Amp^r^	Invitrogen
pZA31 MCS	p15A ori PLtetO-1 Cm^r^	Expressys
pZS21 MCS	pSC101 ori PLtetO-1 Kan^r^	Expressys
pCP20	*bla*, *flp*, temperature-conditional replicon	CGSC #7629
pUC-tesAT	pUC57 harboring *tesAT*	This Study
pUC-tesBT	pUC57 harboring *tesBT*	This Study
pUC-tesBF	pUC57 harboring *tesBF*	This Study
pUC-acc	pUC57 harboring *acc*	This Study
pQE-tesAT	pQE30 harboring *tesAT*	This Study
pQE-tesBT	pQE30 harboring *tesBT*	This Study
pQE-tesBF	pQE30 harboring *tesBF*	This Study
pZA-tesAT	pZA31 harboring *tesAT*	This Study
pZA-tesBT	pZA31 harboring *tesBT*	This Study
pZA-tesBF	pZA31 harboring *tesBF*	This Study
pZS-tesAT	pZS21 harboring *tesAT*	This Study
pZS-tesBT	pZS21 harboring *tesBT*	This Study
pZS-tesBF	pZS21 harboring *tesBF*	This Study
pZS-fadR	pZS21 harboring *fadR*	This Study
pZS-fabZ	pZS21 harboring *fabZ*	This Study
pZS-fadR-fabZ	pZS21 harboring *fadR* and *fabZ*	This Study
pZS-acc	pZS21 harboring *acc*	This Study
**Primers**		
*fadR*_salI_F	GATAGTCGACAAAGAGGAGAAATTAACTATGGTCATTAAGGCGCAAAGC	This Study
*fadR*_claI_R	AGCTATCGATTTAGTGATGGTGATGGTGATGTCGCCCCTGAATGGCTAAATC	This Study
*fabZ*_claI_F	AGCTATCGATAAAGAGGAGAAATTAACTATGTTGACTACTAACACTCATACTCTGC	This Study
*fabZ*_HindIII_R	GCAAAGCTTTCAGTGATGGTGATGGTGATGGGCCTCCCGGCTACG	This Study
phage_*fabR*_F	CGTTCATTCACAATACTGGA	This Study
phage_*fabR*_R	TACCTCTATCTTGATTTGCTT	This Study
Phage_*fadD*_F	CGCATTTTAGAGGTGAAGAA	This Study
Phage_*fadD*_R	GTCTGACGACTGACTTAAC	This Study
Phage_*fadE*_F	ACAAGTAAGGGGCTTTTCG	This Study
Phage_*fadE*_R	GCCTTTCGGCTCCGTTATTC	This Study

Note: the enzyme sites are underlined and homologous regions are in italic.

All chemical reagents were purchased from Sigma Aldrich, Hichem Pvt. Ltd and TCI Chemicals (India) Pvt. Ltd. Kits and enzymes used for molecular biology purpose and their sources were as follows: genomic DNA isolation kit (Qiagen), plasmid miniprep kit (Himedia), PCR and gel purification kit (Genetix), Phusion DNA polymerase (Finnzymes), Taq DNA polymerase (Himedia Pvt Ltd), dNTPs (Fermentas, USA), restriction enzymes and T4 DNA ligase (New England Biolab).

### Plasmid and strain construction

Genes for thioesterases of *A*. *tetradius* (TesAT), *B*. *thetaiotaomicron* (TesBT) and *B*. *formatexigens* (TesBF) and acetyl CoA carboxylase (Acc*) of *Corynebacterium glutamicum* were codon optimized along with C-terminal 6xHis-tag, commercially synthesized and cloned in pUC57 vector by Genscript, USA to obtain pUC-tesAT, pUC-tesBT, pUC-tesBF and pUC-acc plasmid, respectively. To construct the pQE-tesAT/pQE-tesBT/pQE-tesBF, pZA-tesAT/pZA-tesBT/pZA-tesBF, and pZS-tesAT/pZS-tesBT/pZS-tesBF plasmids, the thioesterase genes from pUC-tesAT/pUC-tesBT/pUC-tesBF were digested with *Kpn*I/*Sal*I and subcloned at corresponding restriction sites in pQE30, pZA31MCS and pZS21MCS plasmid, respectively. Similarly, codon optimized gene of acetyl CoA carboxylase (Acc*) from *C*. *glutamicum* was subcloned in pZS21MCS at *Kpn*I/*Cla*I sites to obtain pZS-acc plasmid.

For construction of plasmid pZS-fabZ and pZS-fadR, the *fabZ* and *fadR* genes were amplified from *E*. *coli* MG1655 genome using primer set *fadR*_salI_F/*fadR*_salI_R and *fabZ*_claI_F/*fabZ*_claI_R and cloned at *Sal*I/*Cla*I and *Cla*I/*Hind*III restriction sites in pZS21MCS plasmid, respectively (Table 1). For constructing pZS-fadR-fabZ plasmid, *fabZ* gene amplified using primer set *fabZ*_claI_F/*fabZ*_claI_R was cloned in pZS-fadR at *Cla*I and *Hind*III restriction sites.

### Culture conditions

For small volume cultivation, each strain was inoculated from a freshly transformed single colony on LB agar plate into 5 mL LB medium containing appropriate antibiotics (100 μg mL^-1^ ampicillin, 30 μg mL^-1^ kanamycin or 34 μg mL^-1^ chloramphenicol for cells harboring pQE30, pZS21MCS or pZA31MCS based plasmid, respectively) in culture tube and grown overnight. 1% of this seed culture was used to inoculate 3 mL terrific broth (T.B.) supplemented with 8 g L^-1^ glucose and appropriate antibiotics in culture tube. The cultures were induced with 0.1 mM IPTG in case of pQE30 and 100 ng mL^-1^ anhydrotetracycline in case of pZS21MCS and pZA31MCS expression system when OD_600_ reached to ~0.4 and allowed to grow for an additional 72 hr at 37°C and 180 rpm. Cells were then harvested and used for analyzing butyric acid production.

Butyric acid production in different media was determined by cultivating engineered strains in 5 mL LB medium for overnight as seed culture, 1% of which was used to inoculate different media having 8 g L^-1^ glucose and appropriate antibiotics. Morpholinepropanesulfonic acid (MOPS) minimal medium [21] supplemented with 8 g L^-1^ glucose, 1.32 mM dibasic potassium phosphate, 0.276 mM potassium sulfate, and 9.5 mM ammonium chloride was used with variations depending on nutrient limitation. For nitrogen and phosphorous limitation, the amount of ammonium chloride and potassium phosphate dibasic in the medium was 4.75 mM and 0.240 mM, respectively [22]. M9 and M9-MOPS media were prepared as described earlier [23, 24]

### Western blot analysis

The expression level of the thioesterase genes cloned in different plasmids was confirmed by Western blotting. Briefly, the cultures were grown and induced with appropriate inducer at OD_600_ of ~0.4 and harvested after 4 h post induction. After sonication the soluble cell lysate and pellet obtained were separated on a 12.5% polyacrylamide gel. The proteins were transferred to a nitrocellulose membrane (0.2 μm, Amersham Protran, GE Healthcare Life Sciences), probed with anti–penta-his antibody (H1029, Sigma) followed by HRP conjugated anti-mouse IgG antibody (A4416, Sigma). Color development of the protein bands on blot was performed using DAB (3, 3´-diaminobenzidine tetrahydrochloride) and hydrogen peroxide.

### HPLC analysis

HPLC was used to quantitate glucose, acetic acid, lactic acid, succinic acid, butyric acid, butenoic acid and ethanol. The culture (1 ml) of *E*. *coli* strain was centrifuged at 13,000 rpm for 5 min and the supernatant was filtered through a 0.22-μm syringe filter. The clarified sample was applied to HPLC 1260 Infinity Series HPLC system (Agilent) equipped with an Aminex HPX-87H anion exchange column (Bio-Rad) and refractive index (RI) detector. Filtered and degassed 4 mM H_2_SO_4_ was used as mobile phase at a flow rate 0.3 mL min^-1^. The column temperature was maintained at 40°C in a thermostat chamber. The concentrations of metabolites were quantitatively determined by calibration curve made using standards obtained from Absolute Standards, USA. All results were presented as average and standard deviation of the data from two independent experiments.

### Identification and quantification of free fatty acid

Free fatty acids (FFAs) were analyzed as described previously [15]. Briefly, 0.4 mL of culture supernatant was supplemented with 50 μL of 10% (w/v) NaCl and 4 μL of mixture of 10 mg/mL each of C5, C11 and C17 fatty acids as internal standard. The solution was further acidified with 50 μL of glacial acetic acid followed by addition 200 μL of ethyl acetate. The mixture was centrifuged at 16,000 g for 10 min and esterified with ethanol by adding 900 μL of a 30:1 mixture of ethanol and 37% (v/v) HCl to 100 μL of the organic phase, and incubated at 55°C for 1 hr. The fatty acyl ethyl esters were extracted by adding 500 μL dH_2_O and 500 μL hexane and vortexing for 20 s. The top hexane layer extract (300 μL) was analyzed by GC-MS/MS in full scan mode using Agilent 7890 GC with an Agilent 7000 Triple Quadrupole GC/MS system. The column used was Zebron ZB-5MS (length: 30 m; diameter: 0.25 mm; film thickness: 0.25 μm) and the method included injection volume as 1 μL without split mode and oven temperature ramped from 40 to 325°C (initial ramp at the rate of 5°C per min from 40 to 75°C and then 10°C per min until 325°C) and the total run time was 41 min. Compound identities were determined via GC retention times of known standards (FAEES mix from Sigma Aldrich) and verified by mass spectra using NIST library.

### Enzyme assay

Thioesterase activity was measured spectrophotometrically using the method reported earlier [25] with minor modifications. The reaction mixture for thioesterase contained 60 mM potassium phosphate buffer (pH 7.4), 100 μM 5,5'-dithiobis-(2-nitrobenzoic acid) (DTNB), 0.08 mg bovine serum albumin, 20 μM butyryl-CoA, and crude enzyme from cell extract. Reaction was started with the addition of enzyme. Reduction of DTNB by the CoA liberated in the thioesterase reaction was measured at 412 nm using Ultraspec 3100 pro spectrophotometer (Amersham Biosciences). One unit of enzyme activity was defined as the amount of enzyme catalyzing the cleavage of 1 nmol of acyl-CoA per min under the above conditions. The molar extinction coefficient of reduced DTNB was considered as 13,600 M^-1^ cm^-1^.

### Batch cultivation

Batch cultivation was performed in a 0.5 L stirred bioreactor (Sartorius BIOSTAT® Qplus). Freshly transformed single colony was grown overnight in 5 mL LB medium with 34 μg mL^-1^ chloramphenicol at 37°C and 180 rpm. Secondary culture was prepared by inoculating 1% of primary culture to 30 mL of TB medium supplemented with 8 g L^-1^ glucose and 34 μg mL^-1^ chloramphenicol and grown at 37°C until OD_600_ reached to 2–3. The grown secondary culture was inoculated in a bioreactor with working volume of 350 mL having TB media along with 8 g L^-1^ glucose, 100 ng mL^-1^ anhydrotetracycline and 34 μg mL^-1^ chloramphenicol. To identify optimal aeration conditions, dissolved oxygen (DO) was maintained at 5, 20, 40 and 60% saturation and pH was maintained at 7 via the addition of 2N NaOH through PID (proportional, integral and derivative) controller. Samples were collected at regular interval to analyze growth and metabolite production.

### Fed-batch cultivation

For fed-batch cultivation, cells were grown in 0.5 L stirred bioreactor as described above. The reactor was operated in batch mode for 3 h, following which feed containing 20% yeast extract and 50% of glucose was initiated and feed rate was maintained to keep effective concentration of glucose in the reactor below 5 g L^-1^. Dissolved oxygen (DO) was maintained at 40% saturation and pH was maintained at 7 via the addition of 2N NaOH through PID controller. Samples were collected at different time interval for measuring cell density and metabolite concentration.

For fed batch cultivation in MOPS medium under phosphorous limitation, freshly transformed single colony was first grown overnight in 5 mL LB medium with 34 μg mL^-1^ chloramphenicol at 37°C and 180 rpm. The primary culture (1%) was used to inoculate 300 ml MOPS minimal medium having 10 g L^-1^ glucose without the phosphorous limitation. When OD_600_ reached ~3, this secondary culture was used to inoculate 3 L of same medium in a 5 L bioreactor (Applikon). Upon reaching OD_600_ of ~4, the tertiary culture was centrifuged and the cells were resuspended in 350 ml of MOPS medium with phosphorous limitation (0.53 mM phosphate instead of 1.32 mM) containing 10 g L^-1^ glucose (a C/P ratio of 630 which has been reported best for production of FFAs [22]), 100 ng mL^-1^ anhydrotetracycline and 34 μg mL^-1^ chloramphenicol and grown in a 500 ml bioreactor (Sartorius BIOSTAT® Qplus) under controlled condition as mentioned before. Feed containing 50% glucose and 26.5 mM dibasic potassium phosphate, was started after inoculation and feed rate was maintained to keep the glucose concentration below 5 g L^-1^. Dissolved oxygen (DO) was maintained at 40% saturation and pH was maintained at 7 via the addition of 10% (v/v) NH_4_OH base through PID controller. Samples were collected at different time interval for measuring cell density and metabolite concentration.

## Results

### Selection of thioesterase for production of butyric acid

**Fig 2:**
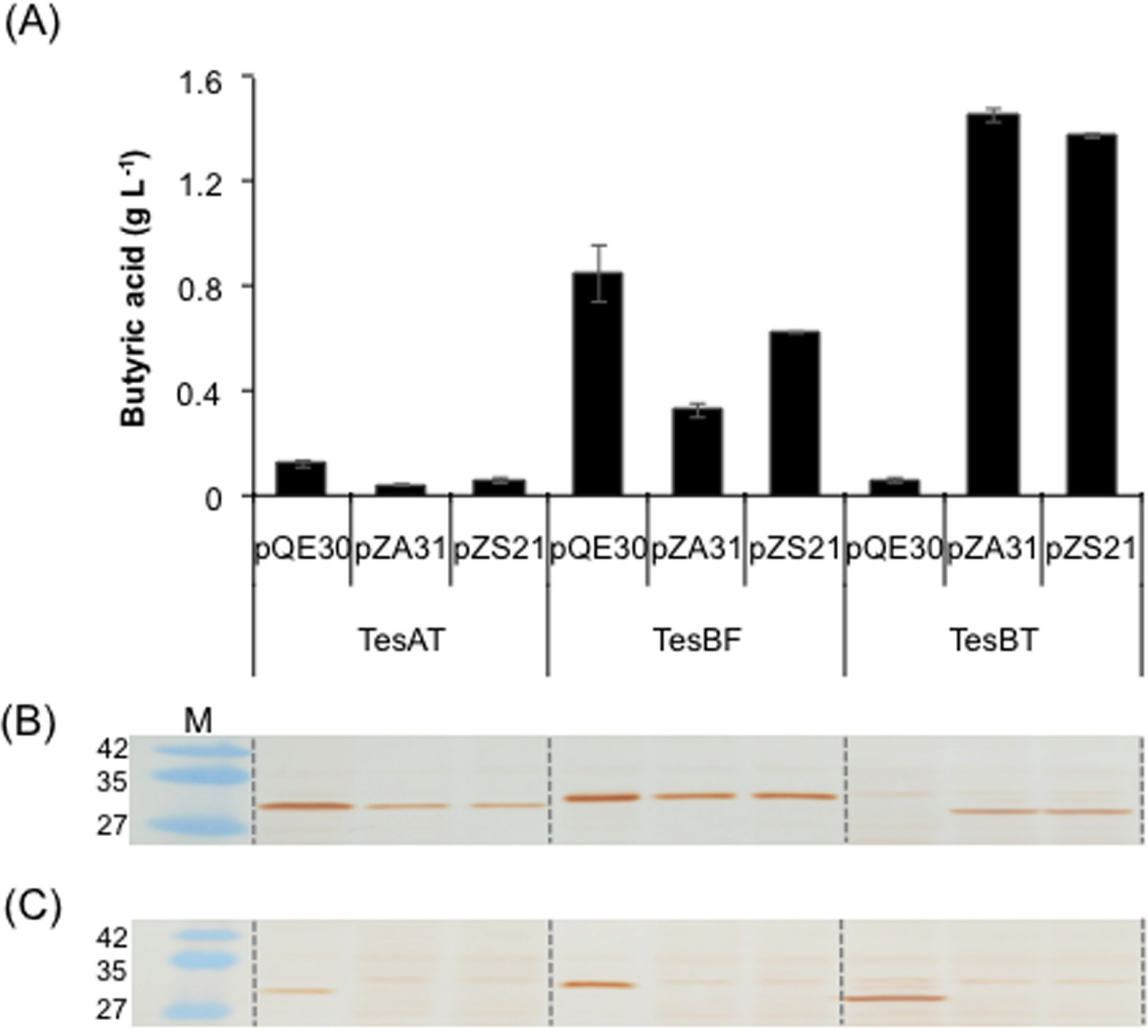
Impact of plasmid type on butyric acid production in *E*. *coli* MG1655 using different thioesterases. (A) Genes for thioesterase TesAT, TesBF and TesBT were cloned in low (pZS21mcs), medium (pZA31mcs) and high (pQE30) copy number plasmid, transformed in *E*. *coli* MG1655 and evaluated for production of butyric acid. Western blot was performed with the soluble (B) and insoluble (C) fraction of the lysate of the cells expressing genes for TesAT, TesBF and TesBT. Protein band of desired molecular weight was observed for each construct in either one or both of the cellular fractions. M indicates protein marker in kilodalton.

We selected three thioesterases, i.e., TesAT, TesBT and TesBF, from *A*. *tetradius*, *B*. *thetaiotaomicron* and *B*. *formatexigens*, respectively, based on their ability to produce SCFAs [18] and synthesized their genes following codon optimization for optimal expression in *E*. *coli*. These genes were further subcloned in three plasmids pQE30, pZA31MCS and pZS21MCS having different copy number (pQE30 > pZA31mcs > pZS21mcs) and transformed in *E*. *coli* to analyze their ability to produce butyric acid. We found marked differences amongst the thioesterases and plasmid copy number used in terms of butyric acid titer obtained. Genes for TesAT and TesBF cloned in high copy number plasmid pQE30 resulted in accumulation of higher butyric acid as compared to medium and low copy number plasmid. However, expression of TesBT in high copy plasmid was detrimental to butyric acid production (Fig 2A). We analyzed expression of these genes on Western blot and found that soluble expression of TesAT, TesBF and TesBT correlated well with the butyric acid titer (Fig 2B and 2C). The TesBT expressed via high copy plasmid mostly accumulated as inclusion bodies (Fig 2C). The data also suggested that since TesAT was expressed well as soluble protein, the lower butyric acid titer in this case was not due to expression problem, but was rather due to lower activity towards butyryl-ACP.

**Fig 3:**
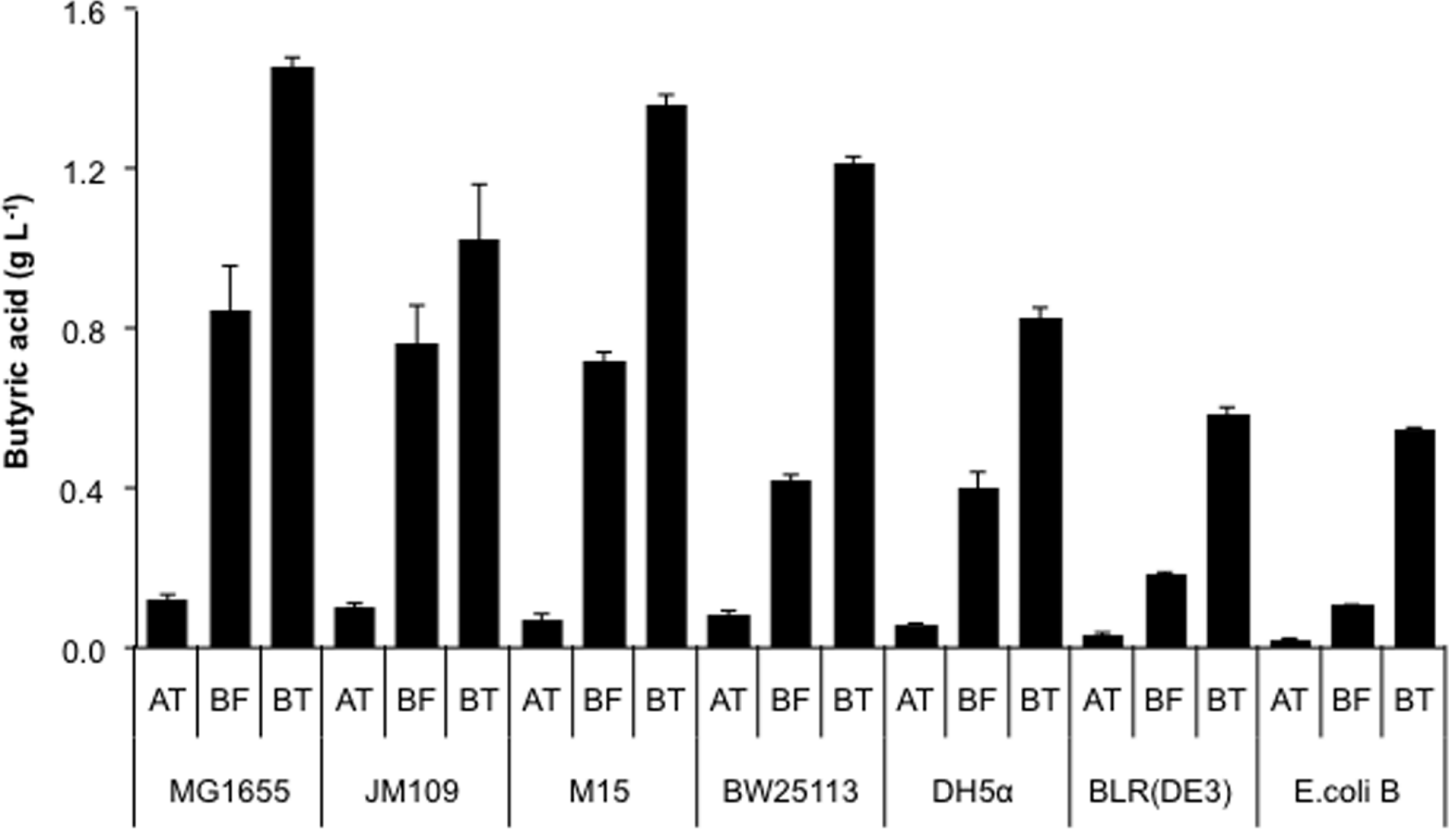
Impact of genotype of *E*. *coli* on butyric acid production using different thioesterases. Different *E*. *coli* strains were transformed with plasmid pQE-tesAT, pQE-tesBF and pQE-tesBT for expression of thioesterase TesAT (represented as AT), TesBF (represented as BF) and TesBT (represented as BT), respectively, and used for butyric acid production.

The genetic background of different *E*. *coli* strains has been found to play significant role in production of biochemicals [19]. We therefore analyzed various *E*. *coli* strains, i.e., MG1655, JM109, M15, BW25113, DH5α, BLR(DE3) and B for production of butyric acid by transforming plasmids containing genes for TesAT, TesBF and TesBT for their optimal expression, i.e., pQE-tesAT, pQE-tesBF and pZA-tesBT, respectively. We found significant variation in butyric acid titer between different strains tested. Among them, MG1655 strain accumulated more butyric acid as compared to other strains when transformed with any of the three thioesterases (Fig 3). However, the titer obtained by transforming plasmid containing *tesBT* or *tesBF* was an order of magnitude higher than that obtained upon transforming plasmid containing *tesAT* for most of the strains (Fig 3). Thus, we selected TesBT and TesBF for our further studies. We screened for optimal inducer concentration and found that 0.1 mM IPTG and 100 ng ml^-1^ anhydrotetracycline were optimal for production of butyric acid in case of TesBF and TesBT, respectively (S1 Fig).

### Chain length specificities of TesBF and TesBT

**Fig 4:**
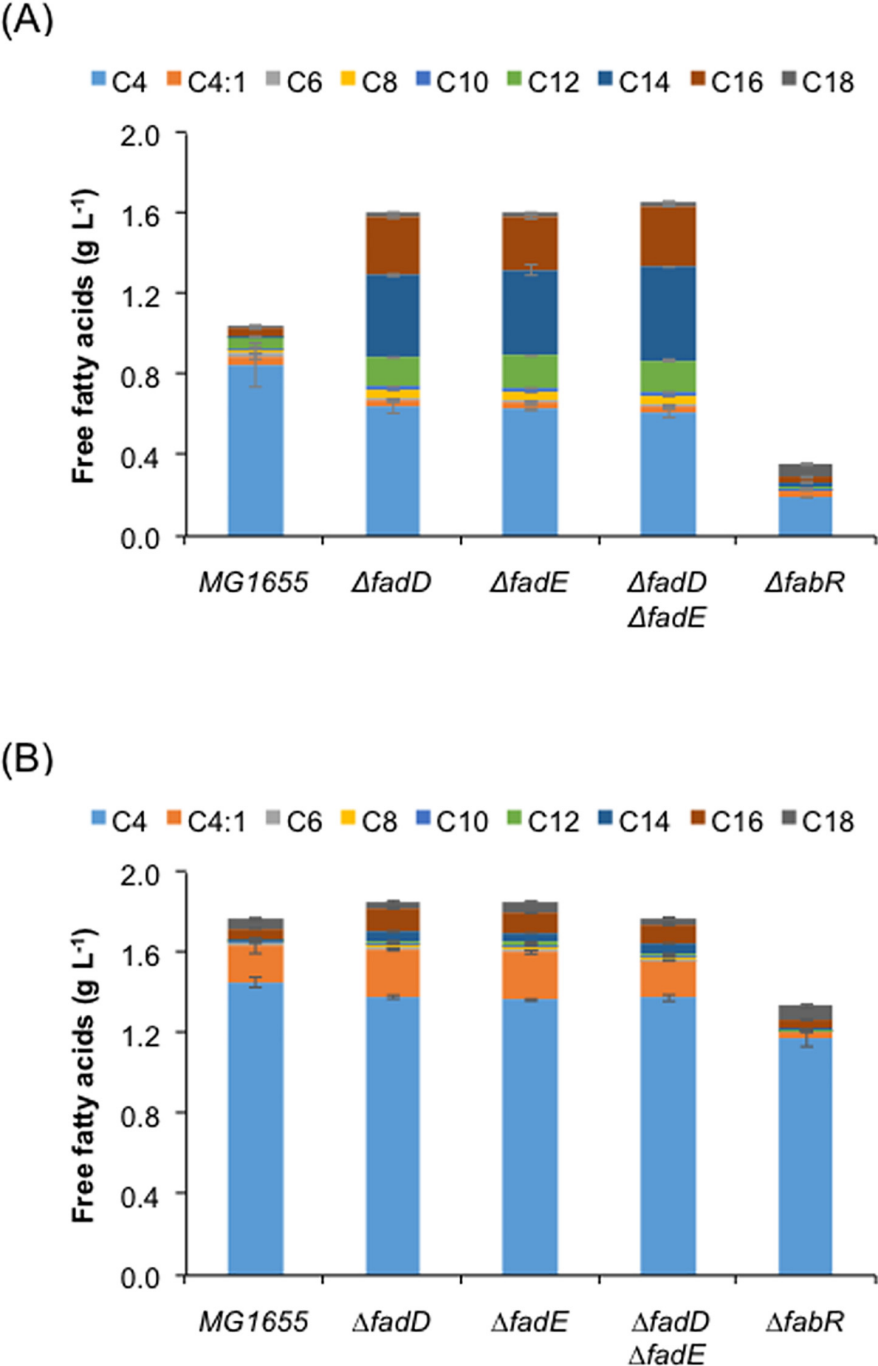
**Impact of deletion of genes having inhibitory effect on host fatty acid biosynthesis on production of free fatty acid using thioesterase TesBF (A) and TesBT (B).** Gene function: *fadD*—fatty acyl-CoA synthetase; *fadE*–acyl-CoA dehydrogenase; *fabR–*transcriptional repressor of FASII.

Complete fatty acid profiling of *E*. *coli* MG1655 transformed with pQE-tesBF and pZA-tesBT showed much higher production of butyric acid as compared to other fatty acids by both TesBF and TesBT (Fig 4). We thus wanted to analyze the chain length specificity of these two enzymes in an *in vitro* enzyme assay. The protein extract of induced culture was used for assessment of activities. TesBT had shown more than two fold higher activities than TesBF towards butyryl-CoA (Table 2). Interestingly we found 2–3 fold higher specific activities of both TesBF and TesBT against longer chain fatty acid precursor of C6 and C8 chain length as compared to butyric acid precursor. Among other intermediates of FASII cycle, activity against malonic acid precursor was negligible, but activity against acetoacetic acid precursor was close to that of butyric acid precursor. TesBF showed low activity against acetic acid precursor, while TesBT showed much higher activity towards it. The reason for higher production of butyric acid under *in vivo* condition could be due to the higher availability of its precursor, i.e., butyryl-ACP, for the thioesterases from FASII pathway as compared to precursors for hexanoic acid or octanoic acid, i.e., hexanoyl-ACP or Octanoyl-ACP. Another important point we noticed was the preference of both TesBF and TesBT for butyric acid precursor as compared to its unsaturated counterpart butenoic acid precursor (Table 2), indicating the reason for observing much higher titer for butyric acid as compared to butenoic acid.

**Table 2:** Chain length specificity of thioesterases.

	Thioesterase	
Substrate	TesBF (nmol min^-1^ mg^-1^)	TesBT (nmol min^-1^ mg^-1^)
Acetyl CoA (C2)	0.11	4.62
Malonyl CoA (C3)	0.00	0.18
Acetoacetyl CoA (C4)	0.76	1.38
Butenoyl CoA (C4:1)	0.43	0.79
Butyryl CoA (C4)	1.28	2.91
Hexanoyl CoA (C6)	2.32	6.63
Octanoyl CoA (C8)	2.06	5.57

### Role of host fatty acid synthesis and degradation pathway on production of butyric acid

Several strategies have been reported in the literature to enhance the production of free fatty acids. Some of these include elimination of β-oxidation, overexpression of key enzymes of the fatty acid biosynthesis pathway and overexpression/deletion of transcriptional regulator. All of these modifications however were tested for production of medium and long chain fatty acids [23, 26–29], and very few attempts have been made for SCFAs production [16].

We thus first tested butyric acid production by knocking out *fadD* (encoding fatty acyl-CoA synthetase) and *fadE* (encoding acyl-CoA dehydrogenase) in order to block β-oxidation cycle (Fig 1). Previously, knocking out *fadD* or *fadE* improved FFAs accumulation [16, 23, 30] as well as fatty alcohols production [31]. However, we observed 25–30% and 5–6% decrease in butyric acid titer in case of overexpression with *tesBF* and *tesBT*, respectively, after deleting *fadD* and/or *fadE* (Fig 4A and 4B). We did observe simultaneous improvement in medium and long chain fatty acids though (Fig 4). This suggested that functional β-oxidation cycle was helping the long chain free fatty acid to get converted to fatty acyl-CoA and further get oxidized into butyryl-CoA. The TesBF or TesBT then was converting butyryl-CoA, in addition to its natural substrate butyryl-ACP, into butyric acid (Fig 1). This observation also supported our previous finding that FadD is not efficient for activation of short chain fatty acid, i.e., butyric acid into butyryl-CoA [19, 32], which otherwise could have led to its subsequent degradation. Thus *fadD* deletion resulted in decrease in butyric acid titer rather than its increase.

**Fig 5:**
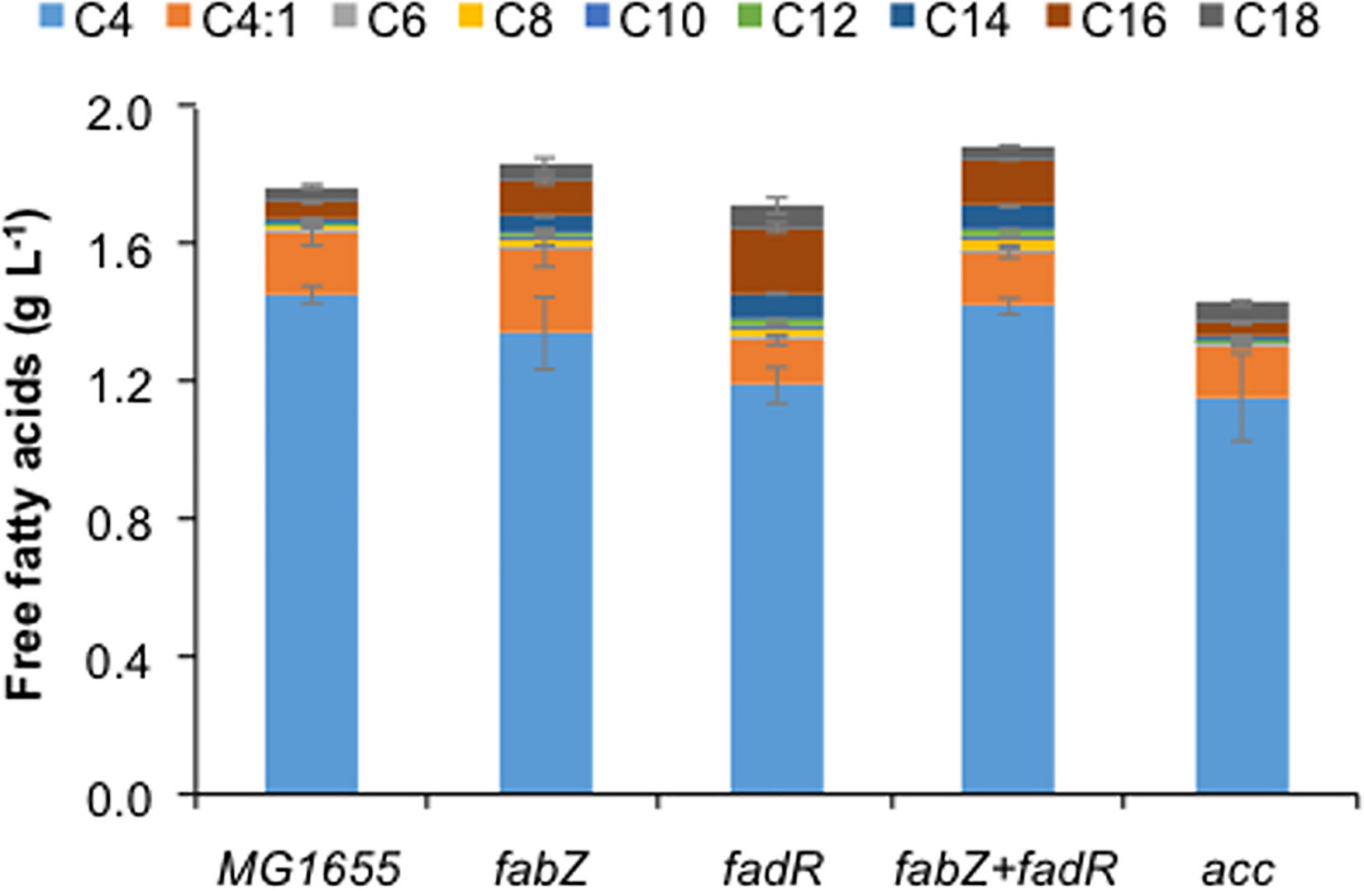
Impact of overexpression of genes having positive effect on host fatty acid biosynthesis on production of butyric acid. *E*. *coli* MG1655 was co-transformed with plasmid containing gene for thioesterase TesBT along with the plasmid containing gene(s) having positive on host fatty acid biosynthesis and analyzed for production of various chain length free fatty acids. Gene function: *fabZ* - β-hydroxyacyl-ACP dehydratase; *fadR*–transcriptional enhancer of FASII and repressor of β-oxidation; *acc*–acetyl-CoA carboxylase of *Corynebacterium glutamicum*.

FabR is a transcriptional repressor which control biosynthesis of fatty acids in *E*. *coli* by repressing expression of the two genes, *fabA* and *fabB*, and its deletion has been shown to improve fatty acid titer and yield, especially for the unsaturated fatty acids [27, 33, 34]. When we checked the effect of deletion of *fabR* on butyric acid production, we found 78% and 20% decrease in butyric acid titer in case of TesBF and TesBT, respectively (Fig 4A and 4B). In order to attempt further improvement in butyric acid titer, we overexpressed *fadR*, a global transcriptional regulator of the fatty acid pathways which controls the expression of several genes involved in fatty acid biosynthesis, degradation, and transport and has been shown to help in improving FFA titer [23]. We also tested overexpression of other genes of fatty acid synthesis pathway on butyric acid production, such as *fabZ* (encoding 3-hydroxy-acyl-ACP dehydratase) from *E*. *coli* and *accABCD* (encoding acetyl-CoA carboxylase) from *Corynebacterium glutamicum*, whose overexpression have been shown to increase free fatty acid titers in *E*. *coli* [35]. We observed enhancement in medium and long chain fatty acid titer in case of *fabZ* and *fadR* overexpression, but all of them showed either no impact or negative impact on butyric acid titer (Fig 5).

### Chemical inhibition of fatty acid elongation to enhance butyric acid production

**Fig 6:**
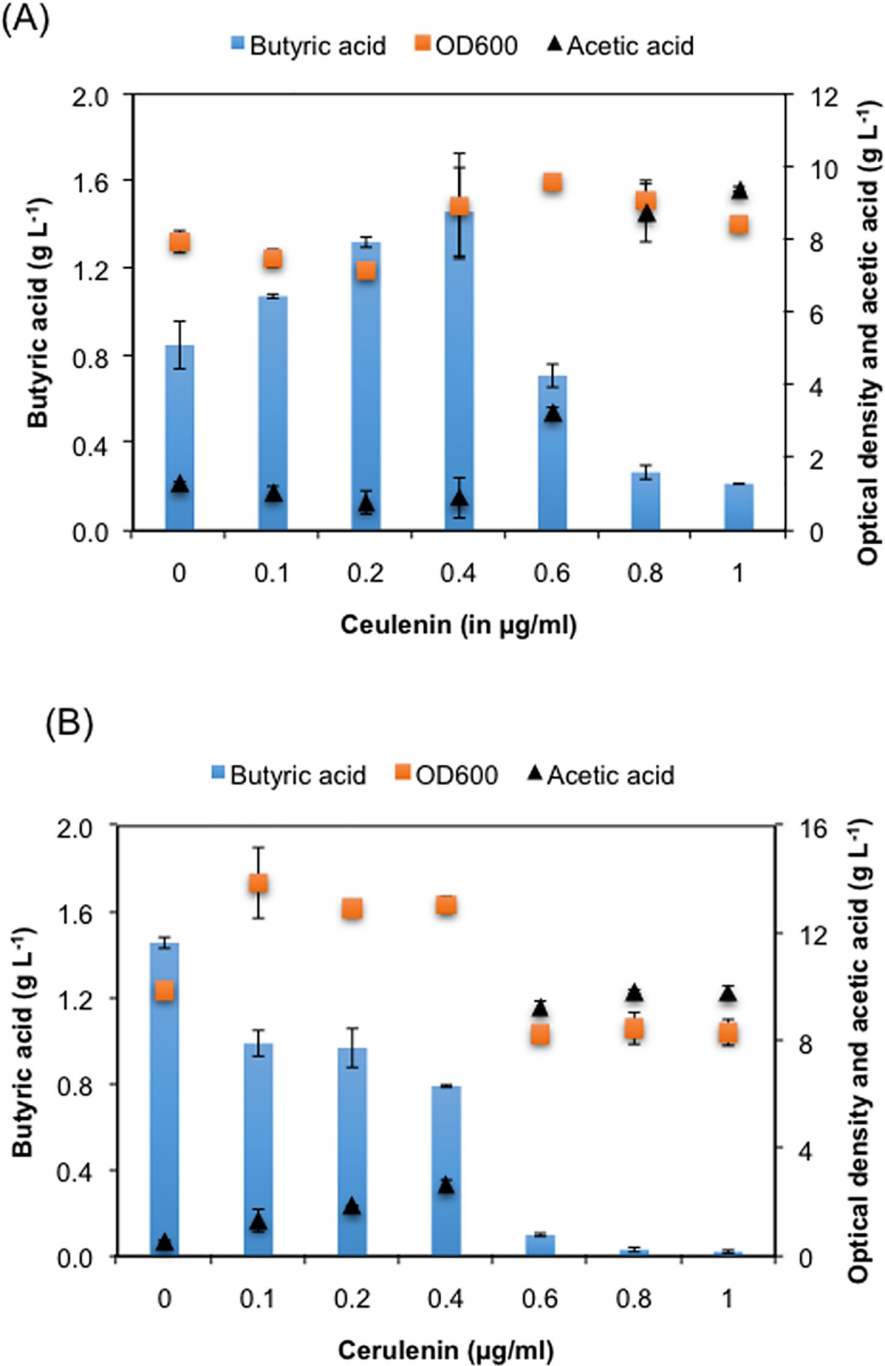
Impact of cerulenin on thioesterase mediated butyric acid production. *E*. *coli* MG1655 transformed with either (A) pQE-tesBF for expression on thioesterase TesBF or (B) pZA-tesBT for expression of thioesterase TesBT were grown in presence of different concentration of cerulenin and extracellular metabolites were analyzed using HPLC.

First cycle of the fatty acid synthesis pathway in *E*. *coli* produces butyryl-ACP, which then enters the next turn of elongation cycle that would decrease the availability of butyryl-ACP for short-chain specific thioesterase. Cerulenin, an antifungal antibiotic, inhibits fatty acid elongation by blocking FabB and FabF and accumulates short chain acyl-ACPs [15, 36]. To test whether addition cerulenin enhances the production of butyric acid, we used different concentrations of cerulenin in the medium and analyzed butyric acid synthesis by the cells transformed with plasmid containing either *tesBF* or *tesBT*. We found diverse impact of cerulenin on butyric acid production depending upon the type of thioesterase used. *E*. *coli* strain where *tesBF* was overexpressed, the butyric acid production increased gradually until 0.4 μg/ml cerulenin added, reaching a titer of 1.46 g L^-1^, which was 1.72 fold higher than the control where no cerulenin was added. The butyric acid titer declined beyond 0.4 μg/ml cerulenin addition, reaching less than 1/4^th^ of the control strain when 1 μg/ml cerulenin was added. On the other hand, cerulenin addition led to decline in butyric acid titer from the beginning when TesBT was used as thioesterase (Fig 6). Notably, acetic acid concentration in the medium showed steep rise at higher concentration of cerulenin complementing with lower concentration of butyric acid (Fig 6). From these observations it appears that cerulenin also inhibits short chain specific thioesterase by occupying its binding pocket as it does with the enzymes of fatty acid elongation cycle. However, inhibitory concentration of cerulenin seems to be different for different thioesterases. To test this hypothesis, we performed *in vitro* enzyme assay of TesBF and TesBT in presence of different concentration of cerulenin. We did find inhibition in enzyme activity upon addition of cerulenin for both TesBF as well as TesBT (Table 3), with varying degree of impact. TesBF seems to be more tolerable towards cerulenin, while gradual decrease in activity was observed in case of TesBT upon increasing concentration of cerulenin, with complete loss of activity beyond 0.6 μg/mL concentration (Table 3). Nevertheless, TesBT showed higher specific activity than TesBF and achieved highest butyric acid titer of 1.46 g L^-1^ without having to add cerulenin (Fig 6). We thus considered TesBT for our further studies to improve butyric acid titer.

**Table 3:** Effect of cerulenin on *in vitro* enzyme activity of thioesterases.

Cerulenin conc. (μg ml^-1^)	Activity of TesBF (nmol min^-1^ mg^-1^)	Activity of TesBT (nmol min^-1^ mg^-1^)
0	1.28	2.91
0.2	1.19	0.99
0.4	1.05	0.46
0.6	0.67	0
0.8	0.56	0
1	0.49	0

### Balancing the cell growth and butyric acid production

After assessing role of host cellular machineries on butyric acid production, we focused on media optimization as media play a key role in energy metabolism of an organism. We envisaged continuous production of butyric acid by keeping the cells in metabolically active but in non-growing state so that substrate can be channeled towards the product formation instead of biomass. Initial experiments done in M9 and MOPS based minimal media using *tesBT* harboring *E*. *coli* strain suggested MOPS being better medium for butyric acid production (Table 4). Further experiments in MOPS based medium showed that the specific yield of butyric acid increased by 2-fold in nitrogen limited medium, but highest titer of butyric acid was recorded in phosphorous limited condition (Table 4). The cultivation of cells in complex medium, i.e., Terrific Broth, yielded lower specific yield, but higher butyric acid titer than the MOPS based minimal medium.

**Table 4:** Impact of media composition and nutrient limitation on butyric acid production.

Media	Cell growth (OD600)	Butyric acid (g L^-1^)	Yield (g g^-1^ glucose)	Specific yield (g g^-1^ cell)
**M9**	6.43 ±0.14	0.02 ±0.0006	0.003 ±0.0001	0.006 ±0.0003
**MOPS**	4.21 ±0.14	0.65 ±0.01	0.081 ±0.001	0.31 ±0.014
**M9-MOPS**	4.38 ±0.14	0.34 ±0.01	0.043 ±0.002	0.16 ±0.015
**MOPS nitrogen limited**	1.96 ±0.01	0.61±0.08	0.085 ±0.001	0.63 ±0.083
**MOPS phosphorous Limited**	3.07 ±0.48	0.77±0.19	0.114 ±0.002	0.50 ±0.047
**T.B.**	9.89 ±0.09	1.45 ±0.03	0.182 ±0.003	0.29 ±0.01

**Fig 7:**
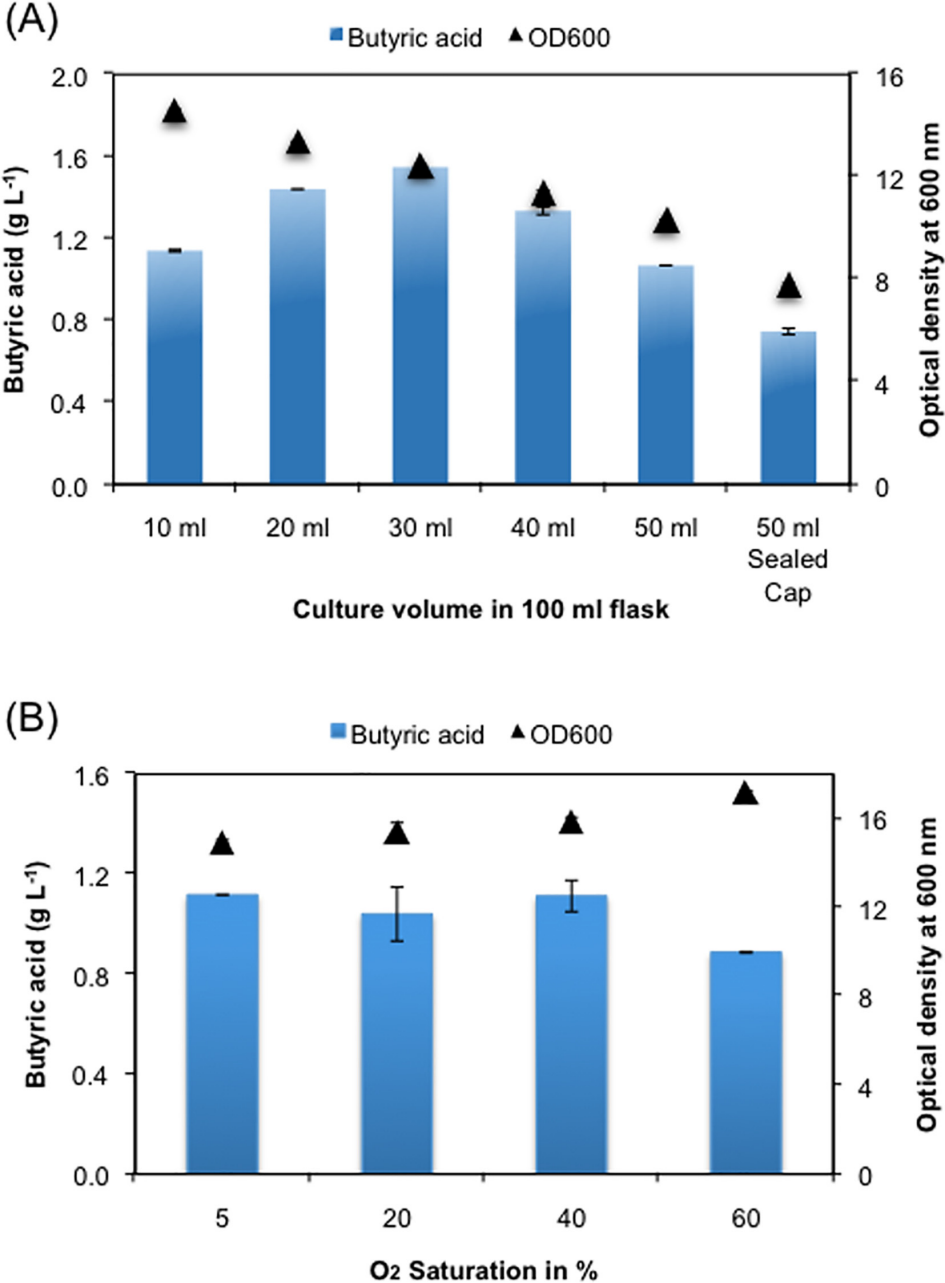
Effect of oxygen availability on production of butyric acid. (A) *E*. *coli* MG1655 transformed with pZA-tesBT plasmid was grown in 100 ml flask containing different volume of culture medium to vary oxygen availability and checked for butyric acid production. (B) *E*. *coli* MG1655 transformed with pZA-tesBT plasmid was grown in the bioreactor containing 350 ml culture medium with different oxygen saturation level and checked for butyric acid production.

Availability of oxygen is a critical parameter for cell growth and metabolite production. We thus tested butyric acid titer by growing different volume of culture medium in a 100 ml flask representing a range of 50–90% of headspace available for oxygen exchange from the air. We also incubated cells in a sealed cap flask for achieving anaerobic growth. Maximum titer of 1.55 g L^-1^ butyric acid was achieved when the liquid to headspace ratio was 3:7 (Fig 7A). The bell-shape profile of butyric acid titer over different headspace suggested a competition of fatty acid precursors for biomass and butyric acid formation. With the availability of high level of oxygen at low liquid to headspace ratio, the flux of FASII cycle is diverted more towards higher biomass formation (Fig 7A) due to active oxidative phosphorylation giving rise to high ATP. At higher liquid to headspace ratio, where growth condition reaching towards anaerobiosis, we again see decline in butyric acid titer due to poor growth rate (Fig 7A). Thus a balance availability of oxygen seems to be important for butyric acid production.

We further tested availability of oxygen on butyric acid production in a bioreactor under batch cultivation. We found that there was not much difference in butyric acid titer (~1.1 g L^-1^) between 5% and 40% saturation level, but butyric acid titer fell to 0.88 g L^-1^ at 60% saturation level with simultaneous increase in cell density (Fig 7B). We therefore selected 40% saturation of oxygen level for further study at fed-batch fermentation level.

### Fed-batch cultivation for high titer butyric acid production

**Fig 8:**
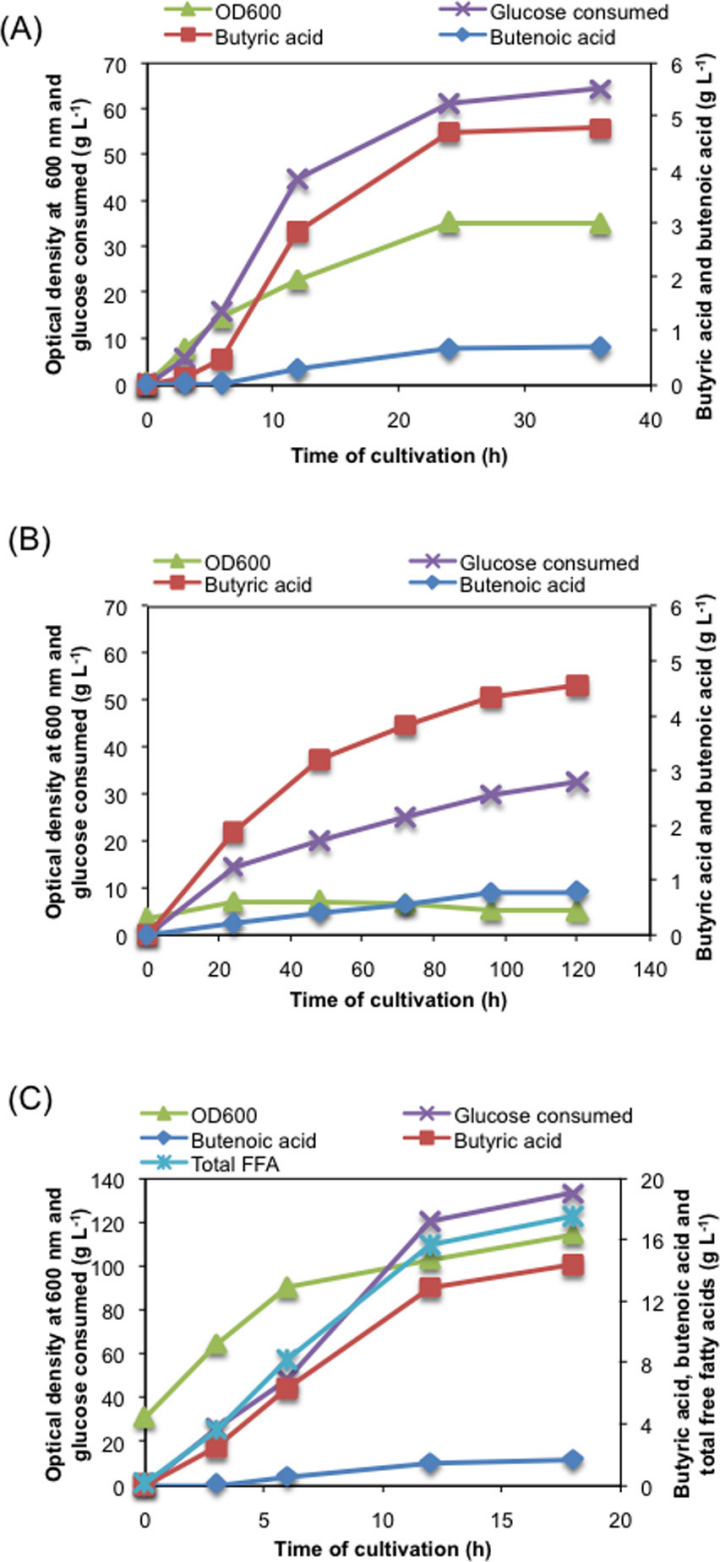
Microbial production of butyric acid in fed-batch cultivation. (A) Fed-batch cultivation performed in Terrific Broth, (B) Fed-batch cultivation performed in MOPS medium under phosphorous limitation, and (C) Fed-batch cultivation performed in MOPS phosphorous limited medium with higher initial cell density.

We performed fed-batch cultivation to increase titer of butyric acid using *E*. *coli* MG1655 harboring plasmid for TesBT production. Use of Terrific Broth (T.B.) at small volume culture in culture tube level resulted in highest titer of butyric acid. We thus made attempt to grow the cells in T.B. containing 10 g L^-1^ glucose and then feed glucose to maintain the glucose concentration at around 5 g L^-1^ in the bioreactor. Cells grew until 24 hr to an OD_600_ of 35 beyond which it entered into stationary phase. Butyric acid titer achieved was 4.77 g L^-1^ and its production seemed to be growth associated as no significant increase in titer was observed beyond 24 hr of growth (Fig 8A). We rather observed significant acetic acid accumulation during later phase of fed-batch cultivation (S1 Table). We further made attempt to use defined medium for butyric acid production in the bioreactor. Since MOPS medium with phosphorous limitation gave best result in culture tube for defined medium (Table 4), we used this medium for cultivation of cells in the bioreactor. The initial glucose concentration in the batch medium was kept at 10 g L^-1^ and feed of concentrated glucose was initiated as soon as glucose concentration went below 2.5 g L^-1^. Residual glucose was monitored at various time intervals and was kept below 5 g L^-1^ in the culture medium by adjusting the feed rate. The butyric acid titer achieved using this strategy was 4.6 g L^-1^ at the end of 120 hr (Fig 8B). To increase the productivity and titer of butyric acid, we inoculated the bioreactor with higher initial OD_600_ of ~30. Most of the butyric acid was produced in the first 12 hr with maximum productivity of 1.29 g L^-1^ h^-1^ (Fig 8C, Table 5). Maximum titer of butyric acid recorded was 14.3 g L^-1^ after 18 hr of cultivation in the bioreactor at 22% of maximum theoretical yield (Table 5). The total free fatty acid detected in the medium was 17.5 g L^-1^ produced at maximum productivity of 1.51 g L^-1^ h^-1^ and product yield of 0.13 g g^-1^ glucose, which was 28% of theoretical maximum (Fig 8, Table 5).

**Table 5:** Yield and productivity of butyric acid during fed-batch cultivation of recombinant *E*. *coli* under phosphorous limiting condition.

Compound	Titer (g L^-1^)	Yield (g g^-1^ glucose)	Molar yield (mM mM^-1^ glucose)	% of Theoretical maxima	Max volumetric productivity (g L^-1^h^-1^)
Butyric acid	14.3	0.11	0.22	22%	1.29
Total free fatty acid	17.5	0.13	0.26	28%	1.51

## Discussion

In this study, we established a biosynthetic pathway for efficient production of butyric acid in *E*. *coli*, which potentially could be used to replace the traditional chemical methods for butyric acid production. We utilized host fatty acid synthesis (FASII) pathway for production of butyric acid with the help of three SCFA specific thioesterases, i.e., TesAT, TesBF and TesBT, and found that TesBF and TesBT overexpression yielded 7 to 12-fold higher butyric acid concentration than TesAT. Genotype of *E*. *coli* strains was shown earlier to have an impact on level of metabolite production [19]. Our result corroborated with this finding as we observed that most of the seven *E*. *coli* strains of different genotype tested showed different results, with MG1655 yielding highest butyric acid titer. Previously, attempts have been made to produce butyric acid in *E*. *coli* through fatty acid synthesis (FASII) pathway by heterologously expressing thioesterases specific for short chain fatty acids (SCFAs) [15, 16, 18]. However, these attempts yielded low butyric acid titer, perhaps due to use of one type of strain, i.e., BL21(DE3). The plasmid copy number also made the difference. The strain MG1655 harboring pZA31-tesBT, a medium copy number plasmid with a PLtetO-1 promoter, achieved the maximum butyric acid accumulation of 1.5 g L^-1^ at culture tube level.

Various reports indicated improvement in medium and long chain fatty acid upon preventing FFA degradation via β-oxidation cycle and by modulating host FASII pathway regulation and elongation. For example, knocking-out initial enzymes of β-oxidation cycle encoded by *fadD* and *fadE* was shown to improve medium and long chain fatty acid accumulation [16, 23, 30]. In contrast, we found decline in butyric acid titer, suggesting positive role of β-oxidation cycle by providing short chain fatty acyl CoA precursor to TesBF and TesBT, which can recognize both ACP and CoA as carrier of short chain fatty acids (Fig 1). Similarly, deletion of transcriptional repressor *fabR* and overexpression of *fabZ* and *accABCD* of fatty acid synthesis pathway were found earlier to up-regulate elongation cycle [29, 35]. However, here also we mostly observed increase in medium and long chain fatty acid titers. Therefore, it seems accelerating the fatty acid synthesis cycle may be beneficial for production of medium and long chain fatty acids, but not for short chain fatty acids.

Since accelerating long chain fatty acid synthesis mostly led to decline in butyric acid titer, we took the strategy of inhibiting fatty acid elongation cycle by adding a chemical inhibitor cerulenin [15, 36]. It had been shown earlier to increase the accumulation of short chain fatty acid [15]. We did see increase in butyric acid titer to a certain extend in case of TesBF, however this increase failed to produce butyric acid beyond 1.46 g L^-1^ that was produced by TesBT without the addition of cerulenin. Cerulenin addition had no impact on butyric acid titer in case of TesBT, possibly due to its inhibitory impact on thioesterases since thioesterases share common binding pocket for substrate with enzymes involved in fatty acid elongation cycle. This was further proven by performing *in vitro* enzyme assay in presence of cerulenin.

Further improvement in butyric acid was achieved by process optimization. Owing to the same pathway for both cell growth and butyric acid production, a balance was achieved between the two by growing the cells in nutrient and oxygen limiting condition. Keeping these factors in mind, a fed-batch cultivation strategy was devised for production of butyric acid in phosphorous and carbon limiting condition. Finally, we obtained 14.3 g L^-1^ of butyric acid and 17.5 g L^-1^ of total free fatty acid, both values being highest reported so far in engineered *E*. *coli* system [3, 8, 10, 15, 37].

## Supporting Information

S1 Fig Effect of different inducer concentration on the production of butyric acid. (A) *E*. *coli* MG1655 harboring pQE-tesBF and (B) *E*. *coli* MG1655 harboring pZA-tesBT.
(PDF)

S1 Table Metabolite profile of *E*. *coli* MG1655 (pZA-tesBT) in TB medium during fed-batch cultivation. (PDF)
