# Supplementary material for: Engineered Production of Short Chain Fatty Acid in Escherichia coli Using Fatty Acid Synthesis Pathway
Source: PLoS One. 2016 Jul 28;11(7):e0160035. doi: 10.1371/journal.pone.0160035 (PMC4965127; doi:10.1371/journal.pone.0160035)
Supplement: S1 Fig — (A) E. coli MG1655 harboring pQE-tesBF and (B) E. coli MG1655 harboring pZA-tesBT. (PDF) [file pone.0160035.s001.pdf]

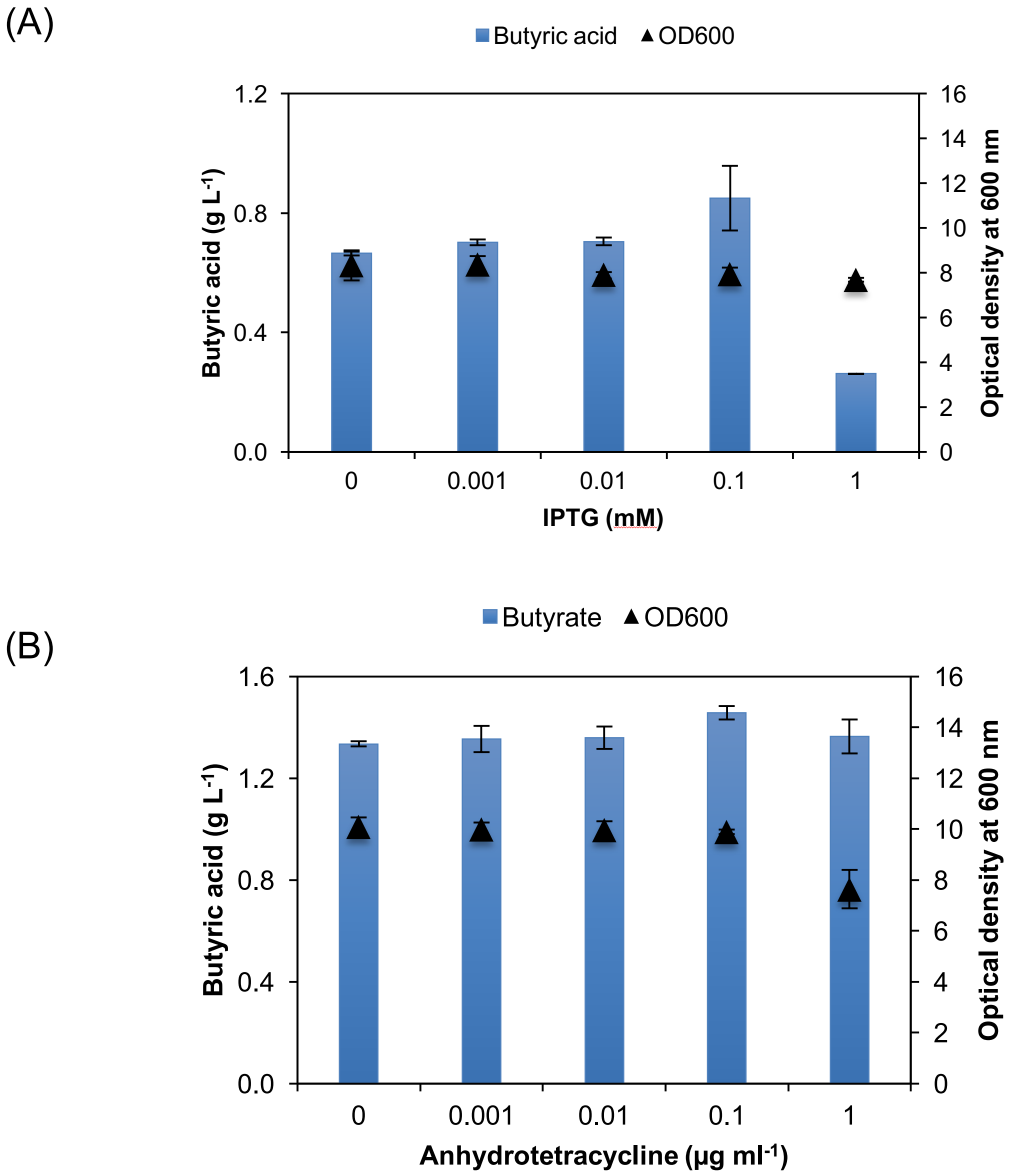

S1 Fig. Effect of different inducer concentration on the production of butyric acid. (A) *E. coli* MG1655 harboring pQE-tesBF and (B) *E. coli* MG1655 harboring pZA-tesBT.
