# Supplementary material for: Engineered Production of Short Chain Fatty Acid in Escherichia coli Using Fatty Acid Synthesis Pathway
Source: PLoS One. 2016 Jul 28;11(7):e0160035. doi: 10.1371/journal.pone.0160035 (PMC4965127; doi:10.1371/journal.pone.0160035)
Supplement: S1 Table — (PDF) [file pone.0160035.s002.pdf]

S1 Table. Metabolite profile of *E. coli* MG1655 (pZA-tesBT) in TB medium during fed-batch cultivation.

**Time (in hrs)**	**0**	**3**	**6**	**12**	**24**	**36**
**Butyric acid (g L^{-1})**	0.02	0.12	0.47	2.85	4.71	4.77
**Butenoic acid (g L^{-1})**	0.02	0.03	0.05	0.28	0.67	0.69
**OD600 (nm)**	0.30	7.74	14.45	22.83	35.25	34.95
**Glucose consumed (g L^{-1})**	0.00	5.53	15.80	44.55	61.00	64.20
**Acetic acid (g L^{-1})**	0.46	3.71	9.13	20.54	28.35	30.49
